# Determination of four arsenic species in environmental water samples by liquid chromatography- inductively coupled plasma - tandem mass spectrometry

**DOI:** 10.1016/j.mex.2020.101183

**Published:** 2020-12-16

**Authors:** Sarah J. Stetson, Caitlyn Lawrence, Susan Whitcomb, Christopher Kanagy

**Affiliations:** a U.S. Geological Survey, Strategic Laboratory Science Branch, P.O. Box 25585, Denver, Colorado, 80225-0585, USA; b U.S. Geological Survey, National Water Quality Laboratory, P.O. Box 25585, Denver, Colorado, 80225-0585, USA

**Keywords:** Groundwater, Surface water, Monomethylarsonate, Dimethylarsinate, Arsenate, Arsenite

## Abstract

Robust and sensitive methods for monitoring inorganic and organic As species As(III), As(V), dimethylarsinate (DMA), and monomethylarsonate (MMA) in environmental water are necessary to understand the toxicity and redox processes of As in a specific environment. The method is sufficiently sensitive and selective to ensure accurate and precise quantitation of As(III), As(V), DMA, and MMA in surface water and groundwater samples with As species concentrations from tens of nanograms per liter to 50 µg/L without dilution of the sample. Mean recoveries of the four species spiked into reagent water, surface water and groundwater and measured periodically over three months ranged from 87.2 % to 108.7 % and relative standard deviation of replicates of all analytes ranged from 1.1 % to 9.0 %.

• A PRP-X100 column and nitrate/phosphate mobile phase was used to separate As(III), As(V), DMA, and MMA in 0.45 µm filtered surface water and groundwater matrices.
• Oxygen was used in the collision cell of the inductively coupled plasma-mass spectrometer with MS/MS mode to shift the measured As mass from 75 to 91.
• The analytical performance of the method and figures of merit including detection limits, precision, accuracy, and interferences when applied to surface water and groundwater matrices were investigated.

## Graphical abstract

**Figure:**
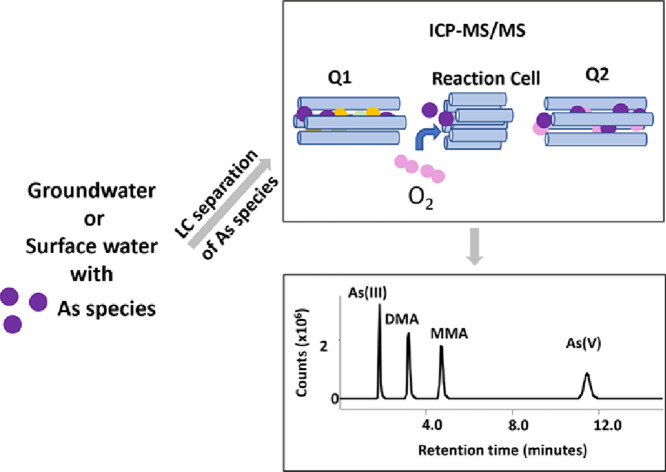

**Specifications Table**

**Table:** 

Subject area	Environmental science
More specific subject area	Water quality
Method name	Arsenic speciation in groundwater and surface water by LC-ICP-MS/MS
Name and reference of original method	Komorowicz I, Baralkiewicz D. Arsenic speciation in water by high-performance liquid chromatography/inductively coupled plasma mass spectrometry-method validation and uncertainty estimation. Rapid Communications in Mass Spectrometry: RCM. 2014;28(2):159-68. Jackson BP. Fast ion chromatography-ICP-QQQ for arsenic speciation. Journal of Analytical Atomic Spectrometry. 2015;30(6):1405-7. Heitkemper DT, Vela NP, Stewart KR, Westphal CS. Determination of total and speciated arsenic in rice by ion chromatography and inductively coupled plasma mass spectrometry. Journal of Analytical Atomic Spectrometry. 2001;16(4):299-306.
Resource availability	NA

## Motivation and background methods

Arsenic species have been measured using a variety of techniques that have been reviewed extensively [1], [2], [3], [4] with many recent methods focusing on chromatographic separation of As species and detection/quantitation of As by inductively coupled plasma-mass spectrometry (ICP-MS) [1,3,5]. When measuring As(III), As(V), DMA, and MMA, anion exchange separation is sufficient for retention and separation because all four species are anionic. Methods that use anion exchange columns, including the Hamilton PRP-X100 column, are widespread [6], [7], [8], and when paired with a nitrate/phosphate mobile phase are promising for achieving good separation of As(III) and DMA, which can be particularly challenging. These methods have been applied to the analysis of As species in rice and reagent water [6,9,10] but method performance in surface water and groundwater matrices has not been previously described. In addition, use of this column/mobile phase pair with an inductively coupled plasma - tandem mass spectrometer (ICP-QQQ) as the As specific detector has not been reported in the literature.

The objectives of the present study are to: optimize the chromatography when using the PRP-X100 column with nitrate/phosphate mobile phase for improved peak resolution of As species; couple the chromatography with ICP-MS/MS detection for improved detection limits when measuring As(III), As(V), DMA, and MMA in 0.45µm filtered surface water and groundwater matrices; characterize the analytical performance of the method; and document analytical figures of merit including detection limits, precision, accuracy, and interferences when applied to surface water and groundwater matrices.

## Reagents, calibrants and quality-control solutions

Ultrapure water from a Barnstead NANOpure system with a resistivity of 18.2 MΩ was used to formulate all reagents and standards. All reagents used were reagent grade or higher purity. Trace metal grade methanol and ammonium hydroxide were used to formulate the mobile phase. Single analyte primary stock standard solutions were purchased from SPEX CertiPrep (Metuchen, NJ) at concentrations of 1000 mg/L (As(III) and As(V)) or 10 mg/L (MMA and DMA). The MMA and DMA standards from SPEX CertiPrep became unavailable for the latter part of the study and new standards were obtained from Absolute Standards (Hamden, CT) at 1000 mg/L. As(III) and As(V) stock standards at 1000 mg/L were purchased from Inorganic Ventures (Christiansburg, VA) for independent calibration verification standards (ICVs). Standard reference material (SRM) 3030 (~20 mg-As/kg as MMA) and SRM 3031 (~20 mg-As/kg as DMA) primary stock standards were purchased from the National Institute for Standards and Technology (NIST) to use as ICVs for MMA and DMA, respectively. Germanium solution (1000 mg/L, SPEX CertiPrep, was spiked into the standards, samples and mobile phase to yield 20 µg-Ge/L.

A mixed intermediate calibration solution that contained all four As species of interest (200 µg/L) and mixed working standards were formulated in 2.5 mM EDTA to match the sample matrix. Working calibration standards formulated by diluting the mixed intermediate calibration solution contained all four As species at seven concentration levels from 0.05 to 50 µg/L (Supporting Information (SI) Table SI-1). The calibration standards at 0.5 µg/L and 5 µg/L were used for continuing calibration verifications (CCVs). ICVs were formulated at 20 µg/L (nominal) in 2.5 mM EDTA. Intermediate and working standard solutions were stored at 4 °C in the dark.

## Instrumentation

Chromatographic separation and detection of the four As species was achieved by ion exchange chromatography using an Agilent (Santa Clara, CA) 1260 Infinity Liquid Chromatography (LC) system that included a Bio-inert pump with degasser, Bio-inert multisampler with Peltier cooled sample tray, a temperature controlled column compartment, and an Agilent 8900 triple quadrupole ICP-MS (Santa Clara, CA). A Hamilton PRPX-100 column with 6-mM ammonium phosphate/ 6 mM ammonium nitrate/ 20 µg/L germanium/ 2% methanol mobile phase, adjusted to pH 6.2 using 20% ammonium hydroxide solution [9,10] was used to separate the four As species. See Table 1 for method parameters and instrument settings. Ge was included in the mobile phase so that its signal could be used for point-to-point internal standard correction of instrument drift. Methanol was added to the mobile phase to improve ionization of As in the plasma [11], [12], [13], [14]. The ICP-QQQ was operated in MS/MS mode with oxygen introduced into the collision/reaction cell to shift As^+^ from mass 75 to mass 91 through the formation of ^75^As^16^O^+^ in the cell. Data were collected in time resolved analysis mode. Point-to-point signal correction was automatically performed in the MassHunter software (Agilent, Santa Clara, CA) using the Ge internal standard signal intensity monitored over the course of the data acquisition. When point-to-point correction is used, every data point measured in the spectrum is corrected by multiplying the counts of each mass at each spectrum by the ratio of internal standard counts in the calibration blank to those in the sample.

**Table 1:** Liquid chromatography and inductively coupled plasma tandem mass spectrometer instrumentation operating conditions.

*Agilent 1260 liquid chromatography system*	

LC analytical column	PRP-X100, 150 mm by 4.6 mm, 5 µm particle size
Guard column	PRP-X100 guard cartridge
Column temperature	25°C
Mobile phase (isocratic)	6-mM ammonium phosphate/6-mM ammonium nitrate/20 µg/L germanium/2% methanol, pH 6.2
Mobile phase flow rate	1.0 mL/min
Sample tray temperature	4°C
Injection volume	50 µL
Total elution time	15 minutes
Pump pressure limit	200 bar
*Agilent 8900 inductively coupled plasma triple quadrupole mass spectrometer*	

RF Power (W)	1550 W
Plasma gas flow rate	15 L/min
Nebulizer gas flow rate	~0.9 L/min (optimized daily)
Peristaltic pump	0.3 rps
Torch sampling depth	8 mm
Integration time – As	1.5 seconds
Integration time – Ge	0.2 seconds
As monitored masses (Q_1_→Q_2_)	75 →91
Ge monitored masses(Q_1_→Q_2_)	72→72

[LC, liquid chromatography; mm, millimeters; µm, micron;°C, degrees Celsius; µg/L, micrograms per liter; mM, millimolar; %, percent; mL/min, milliliters per minute; µL, microliter; W, watts; L/min, liters per minute; rps, revolutions per second; mm, millimeters; As, arsenic; Ge, germanium; Q, quadrupole]

The retention times of the As species in the standards were used to ensure that the correct peak was assigned to each As species. Where the As species concentration was at or above the detection limit, the retention time of each peak was required to be within ±0.5 min of the high calibrator or nearest quality control (QC) sample in order to assign the peak to an As species. Peak resolutions between adjacent peaks for the four As peaks in the 50 µg/L standard measured by the LC/ICP-MS/MS method were calculated according to Eq. 1:

(1) $$resolution\;\left(R\right)=\frac{\;\Delta t_{r}}{w_{av}}$$

where *Δt_r_* is the separation between peaks in units of time and *w_av_* is the average width of the two peaks in units of time.

## As speciation analysis procedures

Just prior to speciation analysis, an aliquot of each sample, standard, or blank sample was transferred to a 1 mL LC vial and Ge was spiked into the vial to achieve a final concentration of 20 µg-Ge/L in order to eliminate the water dip on the Ge chromatogram. A calibration was performed at the start of each analysis batch. A best-fit, linear, inverse weighted, 1/x regression model was chosen for the analysis. The correlation coefficient of the calibrator regression was required to be ≥0.999. The ICV at 20 µg/L was analyzed after each calibration and the measured value was required to be within 20% of the expected concentration to proceed with sample analysis. A CCV at either 0.5 µg/L or 5 µg/L and a reagent-blank were measured after the ICV, at the end of the run and after every ten sample injections during the run. The measured values of bracketing calibration checks were required to be within 15% of the expected concentration and the bracketing blank values were required to be within ± the detection limit. A limit of quantitation standard (LOQ) at 0.1 µg/L was run after calibration and at the end of the run. The measured value of the LOQ was required to be ±50% of the expected concentration. Where any QC was outside of the above limits, the data were not used and most, but not all, samples were rerun after performing cleaning and maintenance of the instrumentation.

## Method validation

Approximately one environmental sample per analytical batch was spiked with a solution containing As(III), As(V), DMA, and MMA in the laboratory just prior to As speciation analysis to yield a final spike concentration of 10 µg/l of each species. The parent sample and spike were analyzed in duplicate and relative percent differences (RPD) between the replicates calculated. Blind blanks consisting of samples of ultrapure water preserved with EDTA (2.5 mM final concentration) submitted through the Quality Systems Branch of the US Geological Survey for As speciation analysis blind to the analyst were analyzed with several batches of samples and spikes (*n* = 14).

### Precision and bias studies

An ultrapure water spike consisting of 40 µg/L of each As species was formulated in 2.5 mM EDTA using the same stock standards as used for preparing calibrators. Two surface water and two groundwater samples were prepared separately for the precision and bias studies as follows: aliquots of two surface water samples and two groundwater samples that had been field filtered through a 0.45 µm filter [15] and stored under ambient laboratory conditions were transferred to opaque polyethylene bottles. Samples were amended with 250 mM EDTA to achieve a final concentration of 2.5 mM EDTA and stored at 4 °C ± 2. The concentration of As species in each sample was measured using the LC/ICP-MS/MS method described above. One surface water was spiked with all four As species to achieve approximate final concentrations of 0.3 µg/L of each As species and a second surface water was spiked with all four As species to achieve approximate final concentrations of 40 µg/L of each As species. One groundwater was spiked to achieve approximate final concentrations of 0.5 µg/L of each As species and a second groundwater was spiked to achieve approximate final concentrations of 40 µg/L of each As species. The exact concentration of each species in the final spiked solution are reported elsewhere [16]. The spike concentrations were used to calculate percent recoveries of each species in the three matrices.

Arsenic speciation concentrations in these spiked surface water and groundwater samples were determined during at least four separate analysis events over a three-month time period in order to characterize method bias and variability. Up to four replicates were measured in a single batch.

### As speciation environmental sample collection and analysis

Surface water and groundwater samples for the surface water and groundwater spike were collected from a variety of locations throughout the United States [16] using the methods described in the U.S. Geological Survey National Field Manual for the Collection of Water-Quality Data [17]. Samples collected for As speciation analysis were filtered in the field using a 0.45 µm filter into 10 mL opaque polyethylene bottles that contained 100 µL of 250 mM EDTA preservative to yield a final concentration of 2.5 mM EDTA in the sample [18, 19]. The concentration of EDTA preservative used was in accordance with the U.S. Geological Survey collection methods [19] and is intended to reduce oxidation/reduction reactions of As in the presence of Fe. In all cases, the EDTA molar concentration exceeded the sum of the molar concentrations of Al, Fe, Mn, Ca, Mg, and Sr (molar excess of EDTA) that is considered necessary for eliminating interconversion of As(III) and As(V). A higher concentration of EDTA must be used where the sum of the molar concentrations of Al, Fe, Mn, Ca, Mg, and Sr is higher than 2.5 M. Bottles were filled to the shoulder with sample. All samples were stored at 4 °C ± 2 until analysis. When field spikes were collected, a second bottle was filled with sample and preserved with EDTA in the same way as the parent sample and then either 100 µL or 500 µL (depending on the historic native total dissolved As concentration) of a solution containing 1,000 µg/L of As(III), As(V), DMA, and MMA was added to the bottle using a digital pipet. Prior to withdrawing sample from the bottle for analysis in the laboratory, each sample bottle was weighed. The empty bottle weight was calculated as the mean weight of five empty bottles; the variability in bottle weight was less than 1%. For each field matrix spiked sample, the mean empty bottle weight was subtracted from the sample bottle weight to determine the volume of sample in the bottle and this volume was used to calculate the field spike recovery. Where the volume of spike solution added exceeded 2% of the total volume, the measured spiked sample concentration was adjusted for the dilution caused by the spike. The final spike concentrations were approximately 10 or 50 µg/L of As(III), As(V), DMA, and MMA.

Methods for sampling and measurement of pH, conductivity, residue on evaporation (ROE), nitrate plus nitrite, dissolved As (As_diss_), and select dissolved trace and major ions are included in the supporting information. As_diss_ was measured by ICP-MS with He in the collision cell on a Perkin Elmer Nexion 350D. Data for these parameters are reported elsewhere [16]. Ranges of each parameter in the samples analyzed (excluding field blanks) are as follows: pH, 6.9 to 9.4; conductivity, 84.0 to 3,978 µS/cm; ROE, 125 to 2,793 mg/L; dissolved iron, <10 to 4,015 µg/L; dissolved manganese, <0.4 to 1,287 µg/L; dissolved calcium, 2.40 to 146 mg/L; dissolved magnesium, 0.5 to 118 mg/L; dissolved sodium, 2.1 to 890 mg/L; dissolved As, <0.1 to 1,977 µg/L; nitrate plus nitrite, <0.04 to 12.7 mg/L.

### Method performance

**Fig. 1:**
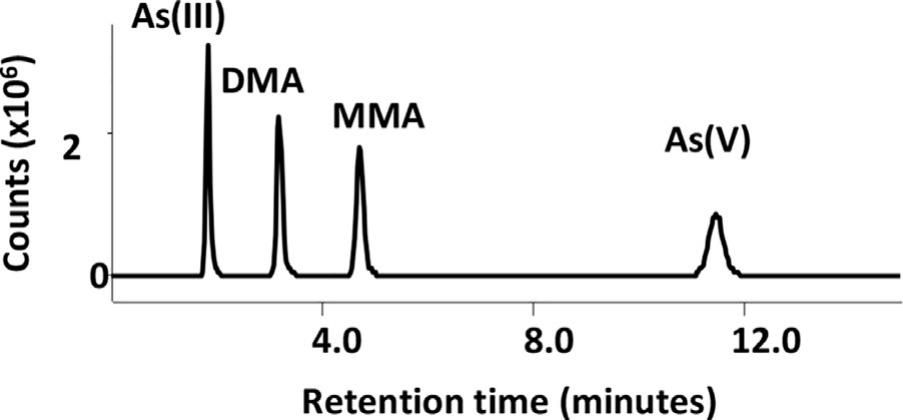
Chromatograph of 50 µg/L arsenic species standard in water using LC/ICP-MS/MS analysis.

All four As peaks were separated chromatographically, with a minimum peak resolution of 2 in the 50 µg/L standard and greater resolution at lower concentrations (Fig. 1). Mobile phase concentrations and pH were optimized to maximize peak separation and total analysis time is approximately 15 min. Slightly faster analysis times can be achieved by adding EDTA to the mobile phase or adjusting the mobile phase pH, but lower peak resolution will be achieved, primarily between As(III) and DMA (data not shown).

The detection limit (DL) was determined using the Environmental Protection Agency spike and blank based procedures [20]. LOQ and blank measurements made over the course of seven months were used in the DL calculation procedure. The blank-based DL was lower than the spike-based DL (Table SI-1). Therefore, spike-based DLs were established to be 0.03 µg/L, As(III); 0.05 µg/L As(V); 0.03 µg/L, DMA; and 0.04 µg/L, MMA [16]. Detection limits, reporting limits, and upper dilution limits are tabulated in Table SI-1. Data between the detection limit and reporting limit were reported as the measured value with a qualifier. Data below the detection limit were reported as less than the reporting limit [21].

Carryover was not observed in the carryover reagent-blank samples analyzed directly following the 50 µg/L calibrator and As was not detected in any of the blind blank samples [16].

### Interferences

There are three general areas where interferences can occur in the analysis of As by ICP-MS: during chromatographic separation of the As species if compounds with m/z of 75 co-elute, signal suppression or enhancement due to variability of ionization in the plasma, and during mass separation of As from other ions in the mass spectrometer. Samples with extreme pH, which were beyond the scope of this study, could potentially cause shifts in the retention times of the analytes or precipitation of the phosphate mobile phase and therefore caution should be used if applying the method to acid mine drainage samples [22, 23].

The primary interference on ^75^As^+^, which is the only stable isotope of As, in this method is the molecular interference of ^40^Ar^35^Cl^+^ formed in the plasma of the MS when chloride is present in the sample. When using a single-quadrupole ICP-MS, a collision cell with helium gas and kinetic energy discrimination is used to remove the ^40^Ar^35^Cl^+^ interference. A second potential source of interference stems from ^150^Nd^2+^ and ^150^Sm^2+^ that also have m/z of 75. It is not likely that either of these ions would co-elute with As during the separation step of the analysis. It is also not common to detect appreciable concentrations of Nd or Sm in surface water or groundwater. However, if present, the single-quadrupole ICP-MS does not remove interferences from ^150^Nd^2+^ or ^150^Sm^2+^ and would represent a positive interference on the analysis. In the ICP-MS/MS analysis, mass 75 is isolated in quadrupole (Q)1 and then transmitted into a reaction cell where As is reacted with oxygen to form AsO^+^. AsO^+^ at mass 91 is then isolated in Q2 and transmitted to the detector. This process removes any interferences that may be present from ^40^Ar^35^Cl, ^150^Nd^2+^, and ^150^Sm^2+^. Blank spikes of HCl, Nd, and Sm were introduced directly to the nebulizer without the LC and analyzed by ICP-MS/MS with oxygen mode. No signal was observed at mass 75, confirming that the interferences are removed when oxygen mode with mass shift is used (Table SI-2).

The Ge IS recovery in the HCl blank spike analyzed by ICP-MS/MS with oxygen mode was 93% indicating that there was no interference from ^35^Cl^37^Cl (Table SI-2). The ^72^Ge^+^ signal for each sample was reviewed to ensure there were no anomalies in the internal standard signal and none were observed, indicating that there were not large signal fluctuations caused by the sample introduction or the plasma during analysis that would cause positive or negative bias in the analysis.

The presence of significant concentrations of other organo-As species in surface water and groundwater samples is not anticipated. However, if present these species could theoretically co-elute with the analytes of interest. Samples from two sites each had three separate unidentified coeluting peaks present with m/z 75. These compounds eluted as shoulders on the As(III) and DMA peaks. Matrix spikes were used to confirm proper peak identification in the presence of co-eluters. The concentrations of the unknown co-eluting species were estimated by comparing the peak areas to those of the nearest eluting known As species and were estimated to represent concentrations within 2 times the DL. If the concentrations of the unknown compounds were higher, some bias could be introduced into the analysis because they elute close to peaks of interest and are not well resolved. It is important to examine each chromatogram for signs of co-eluting peaks that may interfere with the analysis and to maintain retention time criteria that will help to identify anomalies. Maher et al. [24] tabulated several As species that have been found in water, sediment, and tissue. However, without completing a study that includes all As compounds, the co-eluting species cannot be identified. Spiking and dilution of samples with co-eluting peaks can help to confirm a peak as the analyte of interest and better resolve the peaks.

### Method precision and bias in ultrapure water, surface water, groundwater and a reference material

The 0.5 µg/L CCVs that were analyzed as routine QC throughout the method performance studies were included as ultrapure water samples in the precision and bias study along with the ultrapure water spike at 40 µg/L, and the surface water and groundwater spikes. Any spike data from the precision and bias study that were bracketed by QC that did not meet the QC acceptance criteria were omitted from the bias and variability analysis. Therefore, the number of replicates used varies (Table 2). Mean recoveries and precision for each As species spiked in ultrapure water, surface water, and groundwater and measured periodically over a three-month period ranged from 87.2 % to 108.7 % (Table 2). Relative standard deviations of replicates of all analytes ranged from 1.1 % to 9.0% in all matrix spikes (Table 2).

**Table 2:** Precision and bias of four arsenic (As) species in ultrapure water, surface water, and groundwater and limit of quantitation standard (LOQ), continuing calibration standard (CCV) at 5 µg/L, and third-party check standard (ICV).

	Ultrapure water spike/CCV, 0.5 µg/L			Ultrapure water spike, 40 µg/L		
Analyte	n	Mean recovery (%)	RSD (%)	n	Mean recovery (%)	RSD (%)
As(III)	57	102.5	5.1	13	104.8	2.7
DMA	57	102.8	4.6	11	106.7	5.5
MMA	57	103.8	5.1	11	107.0	5.8
As(V)	56	103.0	6.4	11	106.2	4.9
	Surface water matrix spike, 0.3 µg/L			Surface water matrix spike, 40 µg/L		
As(III)	16	97.7	8.7	11	103.1	5.2
DMA	14	99.0	4.8	11	104.1	4.8
MMA	14	103.7	4.8	11	104.7	3.3
As(V)	14	106.2	6.0	10	106.1	3.9
	Groundwater matrix spike, 0.5 µg/L			Groundwater matrix spike, 40 µg/L		
As(III)	15	87.2	9.0	11	104.7	3.6
DMA	13	99.9	4.9	10	102.6	1.9
MMA	13	98.8	4.8	10	103.2	1.1
As(V)	13	108.7	2.3	8	102.8	1.2
	Limit of quantitation standard, 0.1 µg/L			Surface water matrix spike, 40 µg/L		
As(III)	33	97.8	12.3	50	104.2	4.5
DMA	33	95.7	12.2	50	105.1	5.2
MMA	33	96.7	14.5	50	105.1	4.9
As(V)	33	95.4	19.7	50	104.4	4.8
	ICV, 20 µg/L					
As(III)	15	98.2	3.5			
DMA	15	98.8	2.6			
MMA	15	98.7	2.4			
As(V)	15	100.7	4.1			

[n, number of replicates; µg/L, micrograms per liter; %, percent]

Mean recoveries of all As species in the LOQ, CCV, and ICV over a seven-month period ranged from 95.4 % - 105.1 % (Table 2). The relative standard deviations of replicate measurements of each As species in the LOQ (12.2 % to 19.7 %) were higher than in the CCV and ICV samples (2.4 % to 6.4 %), which is expected when concentrations are near the detection limit. Recovery of MMA in NIST SRM 3030 was 104.2 % (*n* = 2) and recovery of DMA in NIST SRM 3031 was 102.0 % (*n* = 2).

### Recovery of As species in laboratory and field matrix spikes

**Fig. 2:**
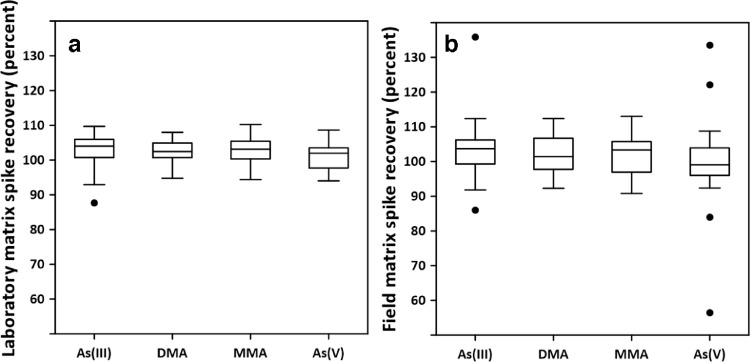
Boxplots of percent recovery of 10 or 50 µg/L As(III), As(V), dimethylarsinate (DMA), and monomethylarsonate (MMA) spiked into surface water and groundwater a) in the laboratory at the time of analysis and b) in the field at the time of collection. Box center line indicates the median, box edges mark the first and third quartiles, whiskers are 1.5 times the interquartile range, and data outliers are points beyond the whiskers.

Most field samples had detectable As(III) and As(V) present. DMA was detected at one site (0.12 µg/L) and MMA was not detected at any site studied. Percent recoveries of laboratory and field matrix spikes at either 10 or 50 µg/L of As(III), As(V), DMA, and MMA measured in groundwater and surface water samples are plotted in boxplots (Fig. 2) and mean recoveries, standard deviation, and number of data points used are tabulated in Table SI-3. The samples spiked in the laboratory and in the field represent a range of pH, conductivity, trace metal, and total arsenic concentrations and are from a variety of locations throughout the United States [16]. The precision and variability of laboratory spike recoveries are comparable to those of the CCV and ICV, indicating that the matrices of the range of surface waters and groundwaters investigated here do not affect the performance of the As speciation method described here.

Field spikes were treated separately from laboratory spikes to assess the variability contributed by the field procedure and sample handling and storage prior to analysis but was not intended to be a full hold-time study. A separate hold-time study is reported [25] which demonstrated that there can be interconversion of As species within 15 days of sampling in certain samples and others can be stable for more than 90 days. There was wider variability in recoveries of field spikes than laboratory spikes (Table SI-3) but mean recoveries were similar. All field spiked samples were subjected to the preservation and storage conditions used in this study (0.45 µm filtration, storage in opaque containers at 4 °C, amendment with a molar excess of EDTA). Isolation of samples from light reduces the possibility of redox processes [26, 27] and filtration reduces the possibility of microbial processes [28], [29], [30], [31]. EDTA is added both for microbial suppression and complexation of metals such as Fe that are involved in As redox processes [18]. Routine use of field spikes is important for understanding any chemical or microbial processes that may be occurring in the sample between collection and analysis. For each As speciation study, regardless of analytical method used, careful attention should be dedicated to ensuring that there is not interconversion of (for example) As(III) and As(V).

### Relative percent difference between duplicates

**Fig. 3:**
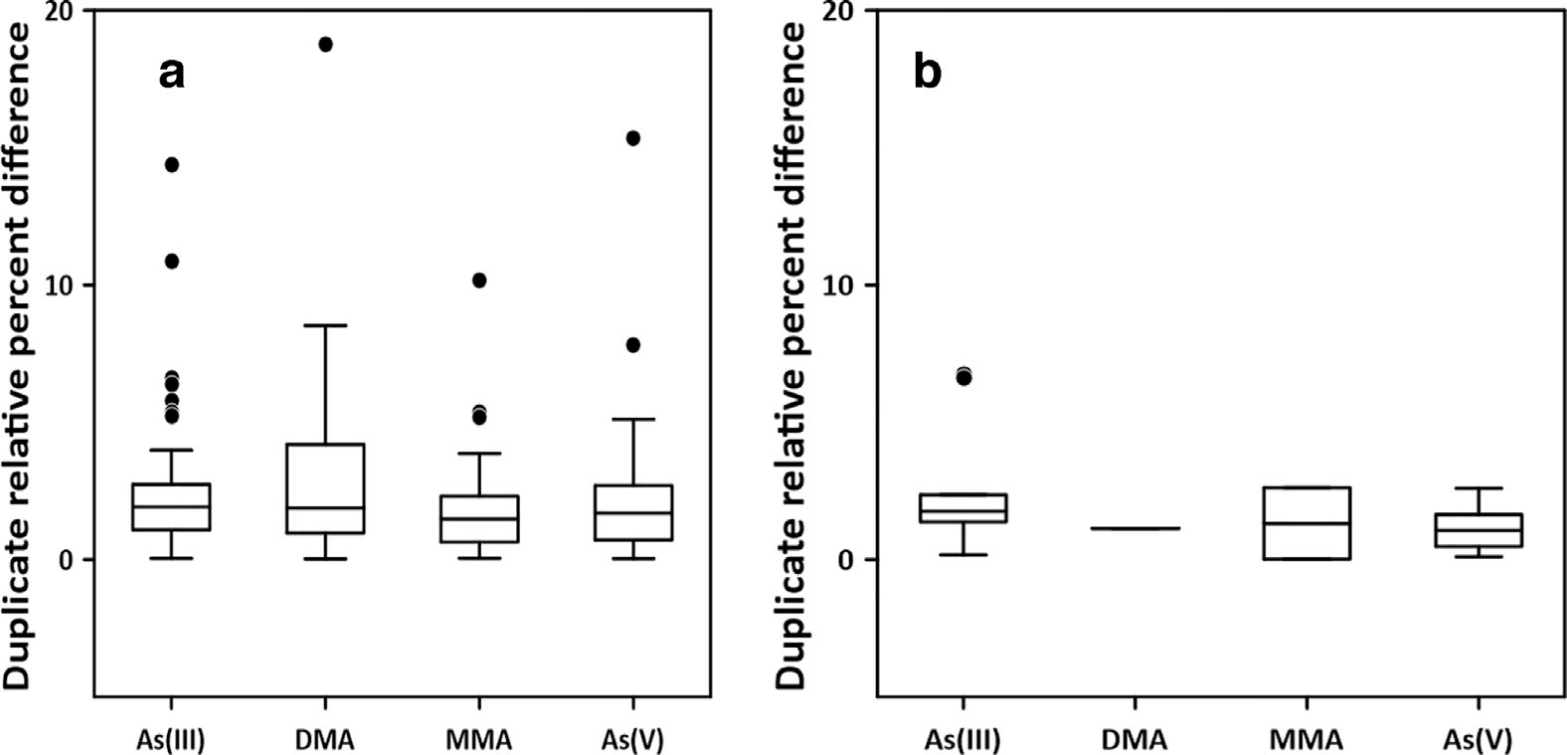
Boxplots of relative percent difference between duplicates for As(III), As(V), dimethylarsinate (DMA), and monomethylarsonate (MMA) that were a) measured in the laboratory on samples and sample spikes or b) duplicate samples collected in the field. Box center line indicates the median, box edges mark the first and third quartiles, whiskers are 1.5 times the interquartile range, and data outliers are points beyond the whiskers.

Relative percent differences for laboratory duplicates, laboratory spike duplicates, and duplicate samples collected in the field are tabulated in Table SI-4 and plotted in Fig. 3. Where one or both values of the sample pair were below the reporting limit [16], the RPD was excluded from the mean calculation. Due to the low concentrations of DMA and MMA in samples, there is insufficient laboratory and field duplicate data for DMA and MMA to derive useful information from the data presented (Table SI-4). Mean RPDs for As(III) and As(V) ranged from 1.1 % to 3.8 % across all types of duplicates (Table SI-4). Similar to As(III) and As(V), the laboratory spike duplicate RPDs for DMA and MMA were 2.3 % and 2.1 % respectively.

### Comparison between speciated As and measured dissolved As

**Fig. 4:**
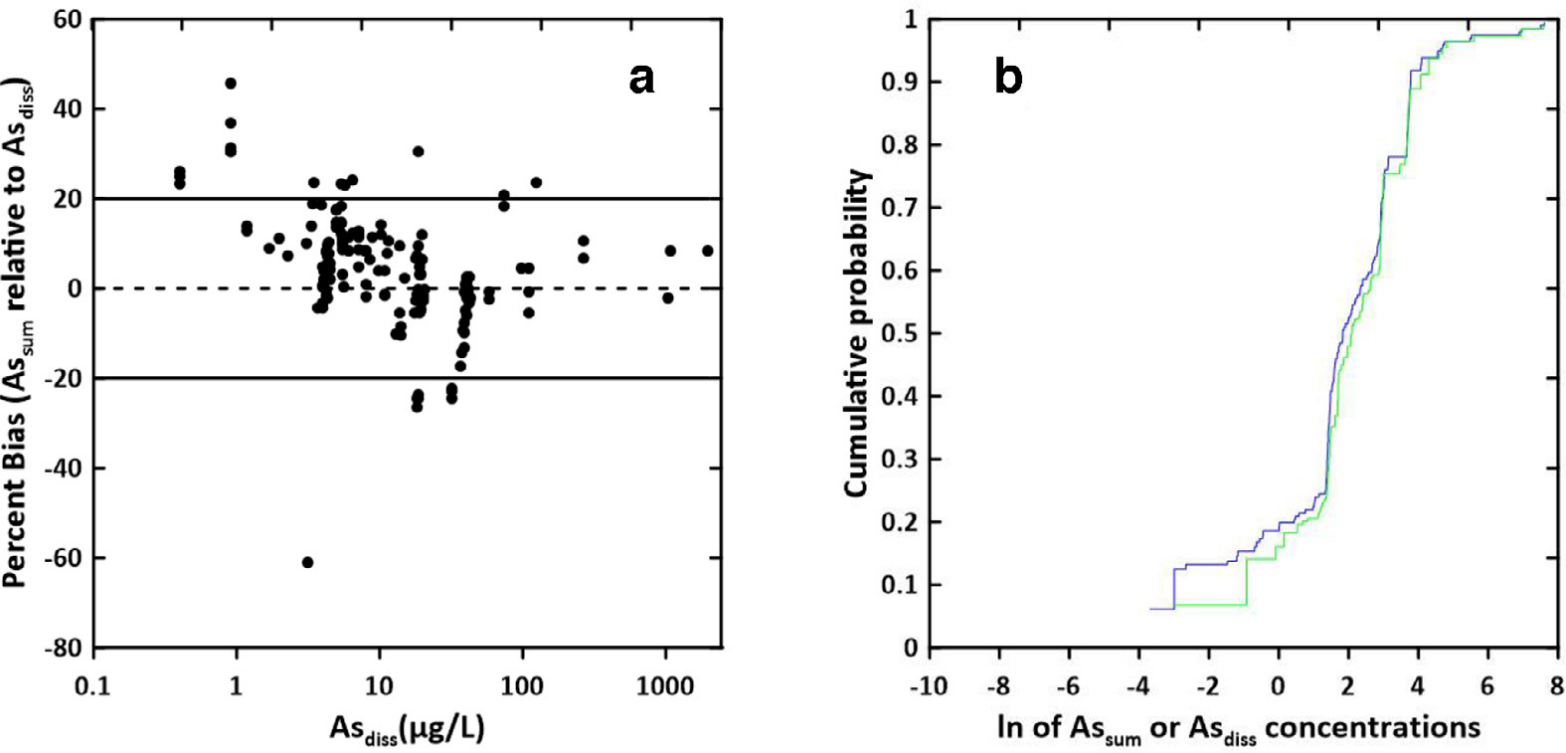
Comparison of the sum of As(III), As(V), dimethylarsinate (DMA), and monomethylarsonate (MMA) arsenic (As_sum_) and total dissolved arsenic measured by inductively coupled plasma mass spectrometry (ICP-MS; As_diss_): a) percent bias of As_sum_ relative to As_diss_ versus As_diss_, and b) cumulative distribution function plots for As_sum_ (blue line, sum of speciation) and for As_diss_ (green line, measured).

The four As species measured by LC/ICP-MS/MS in samples were summed to yield calculated total dissolved As (As_sum_). The sum of the four arsenic species measured using this method was compared to the dissolved As concentration measured using ICP-MS (As_diss_) in order to verify method performance. For statistical analysis of As_sum_ and As_diss_, all data with a value that is less than the reporting limit were replaced with 0.025 (As_sum_) or 0.05 (As_diss_) so that distributions would not be skewed by omitting the censored data in the statistical analyses. A Wilcoxon signed rank test was performed on paired As_sum_ and As_diss_ data using JMP version 14.2 statistical software (SAS Institute, Cary, NC). Cumulative probability distributions were calculated for As_sum_ and As_diss_ using JMP and these data were used to create cumulative probability plots for As_sum_ and As_diss_ (Fig.4).

As_sum_ and As_diss_ data were found to be non-normally distributed by a Shapiro-Wilk Test (*p* < 0.001 for both data sets). A Wilcoxon signed rank test of the paired As_sum_ and As_diss_ data (*n* = 192, *p* < 0.01) indicates that there is a significant difference between As_sum_ and As_diss_. Percent bias was calculated for paired measurements using the formula in Eq. 2.

(2) $$Bias\left(\%\right)=\frac{As_{diss}-As_{sum}}{As_{diss}}\times \;100\%$$

The cumulative probability plots and percent bias versus concentration plot (Fig. 4) show good agreement between As_sum_ and As_diss_ at concentrations above about 10 µg/L. At lower concentrations, As_sum_ is generally lower than As_diss_ and the percent difference between As_sum_ and As_diss_ generally gets larger as As_diss_ decreases (Fig. 4). It is possible that As_sum_ is lower than As_diss_ at low concentrations because the As speciation method is not measuring all possible species of As. While As exists largely in the inorganic forms in most surface waters and groundwaters, metabolites of As or organo-As used as fungicides or antibiotics could be present in water [8] and it is possible that some of them are not retained on the PRP-X column and therefore not detected or quantitated during the speciation analysis. Unidentified As species peaks were observed at concentrations near the DL in samples from one site in Kansas and were also not included in the As_sum_ calculation. While the concentrations of the unidentified species were low, the percent contribution of these or unretained As species to As_diss_ could be significant enough to cause the differences between As_sum_ and As_diss_ observed here.

It is also possible that the molecular or doubly charged overlaps that are an interference on As (most notably ArCl^+^) are not completely removed by the collision cell in He mode during the dissolved As analysis, resulting in high bias in the As_diss_ measurement that is significant at low As concentrations but not at high ones. This cannot be confirmed because the As_diss_ analysis was performed using a single quadrupole instrument. This interference is likely not present in the LC/ICP-MS/MS analysis used for speciation for two reasons: 1) Cl and other ions that form molecular interferences in the plasma of the ICP-MS and doubly charged ions at m/z 75 may be removed or separated from the analytes of interest during the chromatography step and 2) in MS/MS mode, mass 75 is isolated on Q1 and then As is reacted with oxygen in the reaction cell to shift the As signal to mass 91, thereby shifting it away from molecular or doubly charged interferences that could be present (Table SI-2). However, Sm and Nd were not measured in the environmental samples, so it is impossible to know if these elements imparted a positive bias in the As_diss_ analysis.

## Conclusions

The method described here can be used to accurately and precisely measure As species As(III), As(V), DMA, and MMA in surface water and groundwater. The method is sufficiently sensitive and selective to ensure accurate and precise quantitation of As(III), As(V), DMA, and MMA in surface water and groundwater samples with As species concentrations from tens of nanograms per liter to 50 µg/L without dilution of the sample. Anion exchange chromatography was used to achieve good separation between all peaks.

## Declaration of Competing Interest

The authors declare that they have no known competing financial interests or personal relationships that could have appeared to influence the work reported in this paper.

## Appendix Supplementary materials

Image, application 1
