# Supplementary material for: Determination of four arsenic species in environmental water samples by liquid chromatography- inductively coupled plasma - tandem mass spectrometry
Source: MethodsX. 2020 Dec 16;8:101183. doi: 10.1016/j.mex.2020.101183 (PMC7749436; doi:10.1016/j.mex.2020.101183)
Supplement: Supplementary file 1 [file mmc1.pdf]

Supporting Information

Background

Exposure to arsenic (As) in humans, primarily through ingestion or inhalation, can be toxic, mutagenic, and/or carcinogenic (ASTDR, 1999) and inorganic forms of As are generally considered to be more toxic than organic forms (Styblo and others, 2000; Luvonga and others, 2020). Arsenic is present in a variety of groundwaters throughout the world, can be mobilized from geologic deposits into surface water and groundwater sources, and can occur in acid mine drainage (Williams, 2001). In addition to As from geologic deposits, other inputs of As to the environment include historic and current uses of inorganic and organic forms of As as a lumber preservative, pesticide, herbicide, fungicide, and as an antibiotic in livestock production. It is estimated that approximately 2.1 million people use water from domestic wells with As concentrations above the U.S. Environmental Protection Agency's drinking water limit of 10 µg/L (Ayotte and others, 2017). Its toxicity and presence in water sources used for human consumption make it a drinking water quality concern. Robust and sensitive methods for monitoring inorganic and organic As species As(III), As(V), dimethylarsinate (DMA), and monomethylarsonate (MMA), that may be found in surface water and groundwater, are necessary to understand the toxicity and redox processes of As in a specific environment.

ASTDR, 1999, Toxicological Profile for Arsenic (Update). Agency for Toxic Substances and Disease Registry, USDHHS, PHS, Washington DC, https://www.atsdr.cdc.gov/toxprofiles/tp.asp?id=22&tid=3.

Ayotte, J.D., Medalie, L., Qi, S.L., Backer, L.C., and Nolan, B.T., 2017, Estimating the High-Arsenic Domestic-Well Population in the Conterminous United States: Environmental Science & Technology, v. 51, no. 21, p. 12443-12454.

Luvonga, C., Rimmer, C.A., Yu, L.L., and Lee, S.B., 2020, Organoarsenicals in Seafood: Occurrence, Dietary Exposure, Toxicity, and Risk Assessment Considerations – A Review: Journal of Agricultural and Food Chemistry, v. 68, no. 4, p. 943-960.

Styblo, M., Del Razo, L.M., Vega, L., Germolec, D.R., LeCluyse, E.L., Hamilton, G.A., Reed, W., Wang, C., Cullen, W.R., and Thomas, D.J., 2000, Comparative toxicity of trivalent and pentavalent inorganic and methylated arsenicals in rat and human cells: Archives of Toxicology, v. 74, no. 6, p. 289-299.

Williams, M., 2001, Arsenic in mine waters: an international study: Environmental Geology, v. 40, no. 3, p. 267-278.

Table SI-1: Blank detections, detection limit, reporting limit, upper dilution limit and calibrator levels for arsenic speciation method by LC/ICP-MS/MS

[µg/L, micrograms per liter]

Species	Maximum Quality Control Blank Detection (µg/L)	Detection limit (µg/L)	Reporting limit (µg/L)	Upper dilution limit (µg/L)	Calibrator 1 (µg/L)	Calibrator 2 (µg/L)	Calibrator 3 (µg/L)	Calibrator 4 (µg/L)	Calibrator 5 (µg/L)	Calibrator 6 (µg/L)	Calibrator 7 (µg/L)
As(III)	0.01	0.03	0.1	50	0.05	0.1	0.5	1	5	10	50
As(V)	0.04	0.05	0.2	50	not used	0.1	0.5	1	5	10	50
MMA	0.01	0.04	0.1	50	0.05	0.1	0.5	1	5	10	50
DMA	0.02	0.03	0.1	50	0.05	0.1	0.5	1	5	10	50

Table SI-2: Apparent arsenic measured in spikes of neodymium (Nd), samarium (Sm), and chloride (Cl) in oxygen reaction gas mode with mass shift and in helium collision mode by ICP-MS or ICP-MS/MS.
[µg/L, micrograms per liter]

Sample	75 -> 91 As oxygen reaction gas mode (µg/L)	75 As helium collision gas mode (µg/L)	72-> 72 Ge oxygen reaction gas mode (% recovery)	72 Ge helium collision gas mode (% recovery)
Nd 100 ug/L blank spike	0.001	1.95	92.4	100.8
Sm 100 ug/L blank spike	0.002	1.85	92.1	93.7
Blank	0.001	0.001	89.5	93.4
2,000 mg-Cl/L as HCl blank spike	0.009	0.004	93.3	95.8
5,000 mg-Cl/L as CaCl blank spike	0.020	0.31	52.3	57.3

Table SI-3: Mean recoveries, standard deviations (SD), and number of environmental samples spiked and analyzed for arsenic (As) species (n) and spiked either in the laboratory before analysis or in the field at the time of sample collection.

	Laboratory spikes			Field spikes		
	Mean spike recovery (%)	SD (%)	n	Mean spike recovery (%)	SD (%)	n
As(III)	102.6	5.3	25	103.1	9.1	26
DMA	102.4	3.6	25	101.9	5.7	26
MMA	102.3	4	25	101.7	5.6	26
As(V)	101.2	3.8	22	99.6	13.4	24

Table SI-4: Mean relative percent difference (RPD) for each arsenic species calculated from laboratory, laboratory spike, and field duplicates, and the number (n) of pairs used to calculate RPDs.

	Laboratory duplicates		Laboratory spike duplicates		Field duplicates	
	Mean RPD	n	Mean RPD	n	Mean RPD	n
As(III)	3.8	9	2.5	25	2.9	9
DMA	9.9	3	2.3	25	1.1	1
MMA	0.2	1	2.1	25	1.3	2
As(V)	2.5	13	2.1	22	1.1	11
